# Control of the Growth of *Listeria monocytogenes* in Cooked Ham through Combinations of Natural Ingredients

**DOI:** 10.3390/foods12183416

**Published:** 2023-09-13

**Authors:** Gema Nieto, Rocío Peñalver, Carmen Ortuño, Juan D. Hernández, Isidro Guillén

**Affiliations:** 1 Department of Food Technology, Nutrition and Food Science, Veterinary Faculty, University of Murcia, Regional Campus of International Excellence “Campus Mare Nostrum”, Campus de Espinardo, 30100 Murcia, Spain; 2 Cathedra Biotechnology PROSUR, Regional Campus of International Excellence “Campus Mare Nostrum”, Campus de Espinardo, 30100 Murcia, Spainjuandedios@prosur.es (J.D.H.);

**Keywords:** challenge test, *Listeria monocytogenes*, natural ingredients combinations, food safety

## Abstract

In the ready-to-eat food industry, *Listeria* control is mandatory to ensure the food safety of the products since its presence could cause a disease called listeriosis. The objective of the present study was to carry out a challenge test to verify the efficiency of different combinations of natural antimicrobial ingredients against *Listeria monocytogenes* to be used in ready-to-eat foods. Six different formulations of cooked ham were prepared: a control formulation and five different formulations. An initial inoculation of 2 log cycles was used in the different products, and the growth of *Listeria* was monitored at different temperatures and times (4 °C for 17 w and 7 °C for 12 w). Control samples showed a progressive growth, reaching 5–6 log after 3 or 4 weeks. The rest of the samples showed constant counts of *Listeria* during the entire study. Only samples containing 100 ppm nitrite + 250 PPM ascorbic acid + 0.7% PRS-DV-5 did not control the growth of *Listeria* at 7 °C after 7 w of storage. The results obtained allowed us to classify the cooked ham prepared using natural ingredient combinations as a “Ready-to-eat food unable to support the growth of *L. monocytogenes* other than those intended for infants and for special medical purposes”.

## 1. Introduction

Meat products, such as cooked ham, are an important category of processed foods consumed worldwide, classified as ready-to-eat (RTE). These products are highly susceptible to contamination by pathogenic micro-organisms during the production chain due to their physicochemical characteristics and the multiple stages of preparation and handling [1]. The ingestion of this type of food of animal origin does not require additional cooking before consumption, and as such, they are at higher risk of contamination and of becoming sources of contaminated food-borne diseases, being a frequent public health problem worldwide [2]. *Listeria monocytogenes* (*L. monocytogenes*) is a pathogen that is often brought into connection with the safety of RTE products [3]. Despite improved hygiene and production techniques, RTE meat products are still associated with outbreaks of food-borne diseases worldwide [4]. In recent years, outbreaks of food-borne illnesses related to RTE products reported by the Centers for Disease Control and Prevention (CDC) of the United States have been due to infection with *L. monocytogenes* or *Salmonella* (CDC) [5]. *Listeria* is a standard control micro-organism in the food industry as it can be pathogenic and cause a disease called listeriosis, which can affect humans and animals. Cases of listeriosis were linked to deli ham in 2018, deli meats and sliced cheeses in 2019, deli meats (possibly salami, mortadella, and prosciutto) in 2020, and sausages (mortadella, salami, and ham) and sliced cheeses in 2023 [6]. *L. monocytogenes* has a high mortality rate [7]. Due to its facultative anaerobic metabolism, its psychrotrophic properties, and its ability to survive in environmental conditions, this micro-organism cannot only persist in contaminated foodstuffs but also persists in various industrial environments and food contact surfaces (e.g., cutting boards) [8]. In cooked ham, *L. monocytogenes* emerges as the most dominant pathogen, being associated with contamination and cross-contamination throughout the production and processing process [9,10]. This situation is of great concern to the food industry because it is associated with large economic losses and consumer safety. The food industry should produce safe food at reasonable prices using techniques, treatments, or ingredients that assure innocuousness for consumers.

Natural ingredients are increasingly being used as antimicrobial compounds for the development of safe food and are used not only for the concern of consumer health but also for the tendency towards natural food, the so-called “clean labeling”, which has prompted them to be used to replace synthetic additives [11,12,13]. Due to the higher demand for natural origin, chemical-free preservatives, and policies to reduce food waste, fruits and vegetables are being re-evaluated. In this aspect, fruit and vegetable waste has the greatest potential due to its nature, as well as being among the waste categories with the highest production [4]. The fruit and vegetable industry’s waste, such as peel, pomace, pulp, stems, and seeds, contain nutrients (lipids, carbohydrates, minerals, and vitamins) and phytochemical bioactive compounds (pectin, dietary fiber, starch, or phenolic compounds) [11]. The waste generated by the fruit and vegetable industry is agricultural by-products that can be used to obtain commercially valuable bioactive compounds for the development of functional foods due to the large number of health-beneficial attributes related to the by-products consumers have described, including: (i) Antioxidant; (ii) Antimicrobial; (iii) Anti-inflammatory; (iv) Neuroprotective [12,13]. As a result, new trends are aimed at using agricultural by-products as matrices to obtain bioactive compounds of interest to the food industry.

Among natural antimicrobial compounds, a few plant-derived extracts have been extensively studied. For example, the antimicrobial activity of lemon essential oil against *E. coli*, *M. tuberculosis*, and *S. aureus* was investigated, and orange peel essential oil has been used as an antimicrobial against fungi [14,15]. The extracts of citrus commonly used in food receive great attention. In meat and meat products, the citrus extracts can be used alone or combined with other compounds or with a minimum process for a synergistic result [13].

The main objective of the present study is to carry out a challenge test according to the “EURL Lm technical guidance document for carrying out shelf-life studies of *L. mono-cytogenes* in ready-to-eat foods”, to verify the efficiency of different combinations of natural antimicrobial ingredients, against *L. monocytogenes*, to be used in RTE foods, in order to classify these cooked hams as a RTE food capable or not of supporting the growth of *L. monocytogenes*.

## 2. Materials and Methods

### 2.1. Cooked Ham Elaboration and Characterization

Six different cooked ham formulations were manufactured using Good Manufacturing Practices in the Pilot Plant of PROSUR SAU. Each type of cooked ham was made in triplicate.

Ingredients included pork ham meat (80%), potato starch (15 g/kg), sodium tripolyphosphate (5 g/kg), carrageenan (3 g/kg), salt (2% in the final product), and the composition indicated in Table 1.

The cooked ham was prepared according to the following steps: mince the meat using a 16 mm plate; dissolve the dried ingredients into the water to prepare the brine and mix it with the minced meat under vacuum conditions for 1.5 h; stuff the meat in a plastic casing and cook until the core temperature reaches 74 °C; and slice, pack under vacuum conditions, and store at refrigerated temperature. 

The pH of samples was measured using a pH meter (MP220 Basic Mettler Toledo) calibrated with buffer solutions of pH 4.0, 7.0, and 10.0. The water activity (a_w_) was measured at 25 °C ± 1 using a hygrometer (Lab Swift-aw NOVASINA). The tests were performed in triplicate.

### 2.2. PROSUR Ingredients Description

PROSUR ingredients combinations tested in this study are a combination of different dried ingredients: apple cider vinegar and citrus extracts. Dried vinegar has been used as a preservative in combination with citrus extracts, including those that improve its sensory attributes. The primary role of dried vinegar in this combination is to provide antimicrobial properties and inhibit the growth of micro-organisms that cause food spoilage. Meanwhile, the citrus and spice extracts can serve multiple purposes, including enhancing flavor, masking off-flavors, and potentially providing additional antimicrobial properties. This combination can help to prevent the growth of bacteria like *L. monocytogenes* and other spoilage bacteria without any impact on the organoleptic properties of the final product.

### 2.3. Microbiological Analysis 

#### 2.3.1. Choice of Strains and Preparation of the Inoculum

A cocktail of five strains was selected and acquired in the Spanish Type Culture Collection: *Listeria innocua* (*L. innocua*) CECT 8848, CETC 910T, CECT 4030, CECT 5377, and CECT 5378. *L. innocua* strains were used as a surrogate for *L. monocytogenes*, as mentioned in the EURL Guide [16]. The preparation of the inoculum was carried out according to the protocol cited in the EURL guide [16]. Firstly, a pure culture of each strain was inoculated individually in Tryptone Soy Broth (TSB, Pronadisa, Madrid, Spain) at 37 °C and for 24 h. This first subculture was mainly aimed at getting the cells in the stationary phase. Secondly, 100 µL of overnight culture was transferred to a new TSB tube and incubated at a temperature close to the storage temperature of the product (7 °C-7 days, 10 °C-3 days) to adapt the strain to the storage conditions of the product. Thirdly, each second subculture was combined in equal quantity. From the mixed culture, successive dilutions were prepared in buffer peptone water to obtain an inoculum at the expected concentration. The inoculum was used immediately. The targeted inoculum level was checked by enumeration on AL Agar, specific Rapid Chromogenic Media Agar Plates (AL, Agar *Listeria* according to Ottaviani and Agosti, BIO-RAD, (Marnes-la-Coquette, France). 

#### 2.3.2. Preparation and Inoculation of the Test Units

Cooked ham was sliced under sterile conditions. Non-inoculated vacuum packages were prepared for microbiology analysis, containing three slices per package, and were analyzed initially, in the middle, and at the end of the study. Total aerobic counts, lactic bacteria, and *Listeria* were determined to evaluate the initial good practices during the slicing process and a possible *Listeria* contamination. Triplicate non-inoculated samples were analyzed at each analysis time. 

Inoculated samples were prepared with 4 slices per package. An initial microbial concentration of 10^2^ cfu/g was inoculated per slice. Slices were surface inoculated with the cocktail of *Listeria,* and the inoculum was distributed over one surface of each slice and then stacked so that the inoculum was between the slices. Inoculated products were vacuum packaged in gas-impermeable pouches and stored at the appropriate incubation temperature using the guidelines “Parameters for Determining Inoculated Pack/Challenge Study Protocols”, adopted in March 2009 by the National Advisory Committee on Microbiological Criteria for Foods [17] (4 °C or 7 °C). Triplicate inoculated samples were assayed for *Listeria* populations. 

#### 2.3.3. Microbiology Analysis and Storage Conditions

In the non-inoculated samples, total aerobic counts and lactic bacteria were assessed via a spread plating method on specific selective agars, Plate Count Agar (PCA, Condalab, Madrid, Spain), and Man–Rogosa–Sharpe Agar (MRS Agar, Condalab, Madrid, Spain), respectively. Plates were incubated at 30 °C between 48–72 h, before counting. *Listeria* was determined by PCR (PATHfinder-Real-Time PCR Kit for *Listeria monocytogenes* detection, Generon, San Prospero, Italy). Samples were analyzed by triplicating the day 0, in the middle, and at the end of the study.

The inoculated samples were analyzed initially and after 4, 6, 8, 10, 11, 12, 13, 14, 15, 16, and 17 weeks for samples stored at 4 °C, or after 0, 3, 4, 5, 6, 7, 8, 10, and 12 weeks for samples stored at 7 °C [17]. The counts of *Listeria* were assessed via a spread plating method on AL Agar, specifically Rapid Chromogenic Media Agar Plates (AL, Agar *Listeria* according to Ottaviani and Agosti, BIO-RAD, (Marnes-la-Coquette, France). Three independent samples from each temperature were analyzed for each analysis time. Ten grams of cooked ham were homogenized with buffer peptone water in a stomacher for 60 s. 1 mL, and 100 µL of each solution was spread on the specific agar. Plates were incubated for 24 h at 37 °C before counting. 

#### 2.3.4. Growth Potential Parameter

In the present challenge test, based on the “EURL Lm Technical Guidance Document for conducting shelf-life studies on *L. monocytogenes* in ready-to-eat foods”, [16] the growth potential parameter “δ” has been calculated and used for the different cooked ham samples to classify these products:

- When δ > 0.5 log 10 cfu/g, the food is classified into “Ready-to-eat food able to support the growth of *L. monocytogenes* other than those intended for infants and for special medical purposes”;
- When δ < 0.5 log 10 cfu/g, the food is classified into “Ready-to-eat food unable to support the growth of *L. monocytogenes* other than those intended for infants and for special medical purposes”.

Being δ = log_10_ N − log_10_ N_0,_ where N is the number of cells in the sample after the different times of storage, and N_0_ is the initial number of cells just after inoculation. This parameter has been calculated for all the analyzed times, and the most unfavorable data should be used to be able to classify the cooked ham as previously described.

### 2.4. Statistical Analyses

For the microbiological analysis, the results of three slices of each cooked ham were averaged and analyzed for each formulation and sampling day. All means were compared using a one-way analysis of variance (ANOVA) with a 95% confidence level and a Tukey’s multiple comparison test for significantly different means (*p* = 0.05) in SPSS software VS 28 (BM, Armonk, NY, USA).

## 3. Results

The results obtained in the present challenge test are divided into two sections: non-inoculated samples and inoculated samples. 

Regarding the inoculated samples, a physicochemical characterization of pH and a_w_ was carried out initially (Table 1), in the middle, and at the end of the study, but the results are not shown since no differences were observed for any parameter over time throughout the entire study. 

### 3.1. Non-Inoculated Samples

Microbiological analysis of non-inoculated samples was determined for all the cooked ham samples (P1–P6), but only the results obtained for P1 (control sample) are shown in Table 2. The rest of the cooked ham samples did not show counts in non-inoculated samples for any of the analysis times selected, even the analysis of *Listeria* by PCR. For this reason, the results for non-inoculated samples from P2 to P6 are not shown. 

The counts of all the parameters analyzed at time 0 for all the samples were <10 cfu/g, which indicates a good manufacturing practice used during the slicing process. Despite that, a minimal count is normal to have in the sliced samples, which increased during the entire study. This increase was higher in samples stored at 7 °C compared to samples stored at 4 °C, in which the counts at the end of the study were <1 log cycle. No *Listeria* was detected by PCR, with negative results in all cases. This fact suggests a high efficiency of the heat treatment during the production of the cooked ham, an adequate aseptic condition during the slicing process, and that there was no cross-contamination of the inoculated samples. 

### 3.2. Inoculated Samples

#### 3.2.1. Microbial Growth during Storage at Different Temperatures

Table 3 shows the log cfu/g of *L. innocua* for the six types of cooked ham samples at the different study temperatures. The good results obtained in the non-inoculated samples, regarding the *Listeria* results, indicate that the observed growth in the inoculated samples is due to the inoculation using the bacterial cocktail.

Table 3 reveals that the initial microbial inoculation in all the samples was the same, there were no significant differences (*p* < 0.05) between the total cells of *Listeria* inoculated in samples, except for P2, in which the level was slightly lower. It is important to start the challenge test with a similar inoculation level in all the inoculated samples because the evolution of these initial populations will be monitored simultaneously throughout the study.

Sample P1 showed a progressive increase in the growth of *Listeria* at both temperatures. The counts of *Listeria* obtained in the different analysis times were significantly higher (*p* < 0.05) compared to the results of the rest of the samples during the entire study and reached a level of 7.35 log cycles after 17 weeks at 4 °C and 7.76 log cycles after 12 weeks at 7 °C. The statistical analysis showed that the microbial growth obtained after 12 weeks at 4 °C was significantly lower (*p* < 0.05) than those obtained at 7 °C. This fact confirms the importance of maintaining a correct temperature during the shelf life of foods. 

Regarding the results obtained in the rest of the samples, the evolution at 4 °C was very similar in all the samples for the different analysis times. Increasing microbial growth of *Listeria* was not detected in any of the cooked ham samples. In the case of samples stored at 7 °C, the evolution of microbial growth was like those observed at 4 °C for all the different cooked ham products, except in the case of P2 (100 ppm nitrite; 250 ppm ascorbic acid + 0.7% PRS DV-5). Microbial counts of *Listeria* in P2 samples increased progressively, from 1.89 log cycles at time 0 to 3.38 log cycles after 12 weeks. 

A comparison between temperatures has been evaluated for all the samples over a time of 12 weeks. Table 3 shows that only P1 (as explained previously) and P2 showed significantly higher data of microbial growth (*p* < 0.05) in the case of 7 °C compared to 4 °C. However, in the cases of P3, P4, P5, and P6, samples containing the natural ingredients combinations developed by PROSUR, no significant differences were obtained. Therefore, it can be deduced that the use of natural ingredients combinations can reduce the microbial growth of a pathogen, such as *Listeria*, even at 7 °C. These results are important in establishing the maximum shelf life of a product and the maximum temperature of storage allowed, depending on the type of natural ingredients used in the preparation of different types of cooked products. 

#### 3.2.2. Growth Potential Parameter 

The growth potential parameter (δ) has been calculated for all the samples and for all the analysis time using the equation δ = log10 N − log10 N_0_ (Table 4). Table 4 indicates the data for each type of cooked ham. After analyzing all the results obtained, the most unfavorable value (and safest for consumers) was selected to classify the different products, as indicated in the EURL Lm Technical Guidance Document for conducting shelf life studies on *Listeria monocytogenes* in RTE foods.

For any of the temperatures studied, 4 °C and 7 °C, the cooked ham products prepared using any of the natural ingredients combinations developed at PROSUR allowed for a growth potential parameter lower than 0.5 to be obtained; therefore, all of them (P3, P4, P5, and P6) can be classified as a “Ready-to-eat food unable to support the growth of *L. monocytogenes* other than those intended for infants and for special medical purposes”. In the case of cooked ham prepared using nitrates and ascorbic acid (P2), the classification depends on the temperature, and in the case of the control sample (P1), the result is clearly a food able to support the growth of *L. monocytogenes*. 

## 4. Discussion

RTE cooked ham is a product handled before final packaging and is consumed without additional cooking. Possible deterioration and contamination by pathogens during handling can determine the safety of these products during the pre-established shelf life. Antimicrobial compounds provide additional protection to inhibit the growth of microorganisms. This study evaluated the antimicrobial efficacy of different combinations of natural compounds in a real cooked meat product and overcame the negative barriers observed with other natural antimicrobial compounds in ready-to-eat meat products related to fat content or other physicochemical properties of foods [18]. 

The precise handling and preparation of the samples during the challenge test were evaluated using non-inoculated samples, in which microbial growth was only observed in the control samples, being slightly higher in those stored at 7 °C compared to those stored at 4 °C. None of the non-inoculated samples were detected with *Listeria* during the PCR analysis, indicating no cross-contamination of the samples and a clean, sanitary, and safe environment in the pilot plant where the samples were made.

In the food industry, in addition to following strict hygiene and cleanliness standards, the stability and safety of the product must be guaranteed during its shelf life, and antimicrobial ingredients or compounds are required. According to the results obtained for the different cooked ham samples, the antimicrobial properties of the used natural ingredients combinations developed by PROSUR have demonstrated their capacity to inhibit the microbial growth of *Listeria*. As previously described, the natural ingredients are combinations of extracts from citrus and buffered dehydrated vinegar. The antimicrobial activity of a combination of natural food compounds from citrus has been studied previously in different matrices, such as in ranch sauce [19]. The authors obtained a partial fungicidal effect against *Candida metapsilosis* in ranch sauce and an additive antimicrobial effect by combining acetic acid and natural citrus compounds [19]. It opened the possibility of formulating clean label formulations to control spoilage in complex matrices such as sauces.

Kanmani and Rhim [20] investigated grapefruit seed extract, such as antimicrobial in packaging film on *Listeria monocytogenes*, *Bacillus cereus,* and *Escherichia coli*. These authors found a distinctive antimicrobial activity against *L. monocytogenes,* which suggested that the agar containing the grapefruit seed extract can be used in an active food packaging system for maintaining food safety and extending the shelf life of the packaged food. In this case, the final objective was like those followed in the present study: to extend the shelf life of food and to have a safe product for consumers. Along the same lines of including antimicrobial ingredients in packaging films, Zhao et al. [18] investigated the effect of including bioactives, gallic acid, chitosan, and carvacrol in packaging films to control competitive microbiota and *L. monocytoges* in RTE ham products. These authors observed that starch films with gallic acid had the least effect on the antimicrobial activity of ham; however, starch films with chitosan and carvacrol completely inhibited the growth of *L. monocytogenes* during the 4 weeks of storage. The results are in the same line as those obtained in the present study, but it should be noted that the prepared challenge test lasted up to 17 weeks, longer than the time analyzed by Zhao et al [18].

Regarding the use of citrus, Saleem [21] studied the antimicrobial properties of extracts obtained from waste fruit peels of orange and yellow lemon. They used six gram- negative bacteria: *Pseudomonas aeruginosa*, *Klebsiella pneumoniae*, *Serratia marcescens*, *Escherichia coli*, *Proteus vulgaris*, and *Salmonella typhi*, and six gram-positive bacteria: *Staphylococcus aureus*, *Enterococcus faecalis*, *Aeromonas hydrophila*, *Streptococcus pyogenes*, *Listeria monocytogenes*, and *Lactobacillus casei.* The extracts were obtained with different solvents: methanol, ethyl acetate, ethanol, and distilled water. The highest inhibition areas were obtained using distilled water, such as solvent, 22 and 28 mm of inhibition in the case of *L. monocytogenes*, using orange and yellow lemon extract, respectively, measured by the agar well diffusion method. 

The antimicrobial activity observed in the present study using natural ingredients based in vinegar, citrus from lemon, orange, and grapefruit can be related to their content in organic acids, flavonoid, and terpenoids. As has been reported previously [14], the oils from citrus are secondary metabolites that are highly enriched in terpenes. When these compounds contain elements such as oxygen, they are termed terpenoids. The antimicrobial action of terpenes is speculated to involve membrane disruption by the lipophilic compounds [14]. Many food scientists have found the terpenoids present in essential oils of plants to be useful in the control of *Listeria monocytogenes* [22]. Along the same lines, Bakir et al. [23] investigated the antibacterial activity of different vinegars against *Staphylococcus aureus*, *Salmonella Typhimurium*, and *Escherichia coli.* They concluded that the antimicrobial activity observed could partly be related to their acetic acid contents and to their phenolic contents. Concerning the flavonoid content, the antimicrobial activity of these compounds is one of the most important properties reported [18]. The mechanisms attributed to these antimicrobial characteristics have been attributed to different factors that can occur simultaneously or separately, such as the inhibition of the nucleic acid synthesis in bacteria, cell membrane damage, and the inhibition of efflux pumps [24,25,26]. The described mechanisms have a common consequence: the cells’ death.

Regarding the effect of temperature observed during the study, the results are like those previously obtained by other authors, where the temperature of 4 °C helped to maintain the microbial growth of *L. monocytogenes,* compared to 7 °C in different matrices such as salads [27]. It should be noted that in the present study, the different microbial growth was observed only for non-inoculated samples and for P2 samples. The combinations of natural compounds developed at PROSUR and included in the formulation of the RTE cooked ham (P3–P6) made it possible to obtain safe products even though they were stored at 7 °C throughout their useful life.

Furthermore, it is worth highlighting the physicochemical stability of the samples for 14 weeks or 17 weeks in samples stored at 7 °C or 4 °C, respectively, since no significant differences were observed for the pH or water activity parameters. This fact indicates that the use of the combinations of natural compounds developed at PROSUR did not alter the physicochemical characteristics of the elaborated RTE cooked ham.

## 5. Conclusions

The current study has demonstrated that using different combinations of natural ingredients in combination with vinegar, in their different options (high solubility, low salt, emulsion), in the formulation of cooked ham allows that these products be classified as RTE food unable to support the growth of *L. monocytogenes* other than those intended for infants and for special medical purposes. The use of these natural ingredients combinations offers advantages to consumers and the food industry. Their utilization gives the possibility to obtain safer products with a longer shelf life, reducing food waste and economic losses. Additionally, using these natural ingredients combinations avoids the use food synthetic antimicrobial ingredients, allowing a clean labelling on the final product. 

## Figures and Tables

**Table 1:** Description of compounds included in the different cooked ham and their identification. Physicochemical characterization of the samples: pH value ± standard deviation (SD) and a_w_ ± SD measured over time 0 days.

Sample	Preservative Content	pH	a_w_
P1	Negative Control	5.92 ± 0.01	0.91 ± 0.01
P2	Celery (100 ppm nitrite); 250 ppm ascorbic acid + 0.7% PRS DV-5	6.12 ± 0.01	0.91 ± 0.01
P3	1% NATPRE T-10 DV HS + 0.5% PRS-DV-5	6.05 ± 0.04	0.91 ± 0.01
P4	1% NATPRE T-10 DV LS + 0.5% PRS-DV-5 LS + 1.3% NaCl + 0.35–0.40% KCl	5.88 ± 0.02	0.91 ± 0.01
P5	1% NATPRE T-10 EML + 0.5% PRS-DV-5	5.84 ± 0.02	0.92 ± 0.01
P6	1% NATPRE T-10 EML + 0.75% PRS-DV-5	5.87 ± 0.03	0.91 ± 0.01

NATPRE: a combination of extracts from lemon, orange, and grapefruit; HS: high solubility; LS: low-salt content; EML: emulsion; DV: dry vinegar.

**Table 2:** Microbial counts of non-inoculated samples for P1 cooked ham (negative control) storage at 4 °C and 7 °C, initially, in the middle, and at the end of the study.

Storage Temperature (°C)	Analysis Time (Days)	PCA ^1^ (cfu/g)	MRS ^2^ (cfu/g)	PCR ^3^
7	0	<10	<10	Negative/25 g
	7	2.35 × 10^3^	3.16 × 10^2^	Negative/25 g
	12	2.00 × 10^6^	2.43 × 10^4^	Negative/25 g
4	0	<10	<10	Negative/25 g
	9	6.67 × 10^1^	3.17 × 10^2^	Negative/25 g
	17	<10	<10	Negative/25 g

^1^ PCA: count of total aerobic count; ^2^ MRS: count of lactic bacteria; ^3^ Detection of *L. monocytogenes*.

**Table 3:** *Listeria* microbial counts (cfu/g ± SD) of inoculated samples for the different cooked ham samples (P1–P6) stored at 4 and 7 °C at different analysis times.

Temperature	Week	P1	P2	P3	P4	P5	P6
4 °C	0	2.04 ± 0.10 ^a^	1.89 ± 0,11 ^b^	2.28 ± 0.21 ^a^	2.24 ± 0.18 ^a^	2.24 ± 0.28 ^a^	2.57 ± 0.28 ^a^
	4	5.69 ± 0.17 ^a^	1.68 ± 0.29 ^b^	1.77 ± 0.28 ^b^	1.71 ± 0.18 ^b^	1.74 ± 0.31 ^b^	1.93 ± 0.20 ^b^
	5	5.05 ± 0.49 ^a^	1.53 ± 0.21 ^b^	1.74 ± 0.28 ^b^	1.80 ± 0.17 ^b^	2.03 ± 0.38 ^b^	2.26 ± 0.23 ^b^
	7	5.60 ± 0.27 ^a^	1.90 ± 0.10 ^b^	1.82 ± 0.11 ^b^	1.84 ± 0.10 ^b^	1.96 ± 0.23 ^b^	2.11 ± 0.10 ^b^
	8	5.96 ± 0.61 ^a^	1.72 ± 0.24 ^b^	1.92 ± 0.15 ^b c^	1.89 ± 0.30 ^b c^	2.14 ± 0.30 ^b c^	2.36 ± 0.13 ^c^
	10	6.11 ± 0.29 ^a^	1.75 ± 0.18 ^b^	2.08 ± 0.13 ^b c^	1.97 ± 0.07 ^b c^	2.11 ± 0.15 ^b c,^	2.34 ± 0.10 ^c^
	12	5.53 ± 0.15 ^a, 1^	2.03 ± 0.14 ^b, 1^	1.93 ± 0.08 ^b, 1^	2.01 ± 0.09 ^b, 1^	2.04 ± 0.04 ^b c, 1^	2.40 ± 0.22 ^c, 1^
	13	5.39 ± 0.09 ^a^	1.77 ± 0.07 ^c^	1.63 ± 0.13 ^c^	1.86 ± 0.07 ^c^	2.32 ± 0.02 ^b^	2.25 ± 0.25 ^b^
	14	6.00 ± 0.38 ^a^	1.72 ± 0.16 ^b^	1.70 ± 0.12 ^b^	1.84 ± 0.13 ^b^	2.06 ± 0.21 ^b^	2.25 ± 0.25 ^c^
	15	7.60 ± 0.83 ^a^	1.78 ± 0.27 ^b^	1.80 ± 0.21 ^b^	1.78 ± 0.18 ^b^	2.15 ± 0.14 ^b^	2.37 ± 0.28 ^b^
	16	7.36 ± 0.70 ^a^	1.60 ± 0.35 ^b^	1.94 ± 0.42 ^b^	1.73 ± 0.45 ^b^	1.98 ± 0.20 ^b^	2.10 ± 0.15 ^b^
	17	7.35 ± 0.57 ^a^	1.50 ± 0.32 ^b^	1.76 ± 0.33 ^b^	1.81 ± 0.20 ^b^	2.14 ± 0.25 ^b^	2.44 ± 0.11 ^b^
7 °C	0	2.04 ± 0,10 ^a^	1.89 ± 0,11 ^b^	2.28 ± 0.21 ^a^	2.24 ± 0.18 ^a^	2.24 ± 0.28 ^a^	2.57 ± 0.28 ^a^
	3	6.48 ± 0.04 ^a^	2.17 ± 0.30 ^b^	1.93 ± 0.10 ^c^	1.93 ± 0.20 ^c^	2.29 ± 0.22 ^b^	2.46 ± 0.11 ^b^
	4	6.64 ± 0.45 ^a^	2.10 ± 0.19 ^b^	1.85 ± 0.20 ^b^	1.94 ± 0.30 ^b^	2.01 ± 0.52 ^b^	2.16 ± 0.30 ^b^
	5	7.03 ± 0.21 ^a^	2.23 ± 0.24 ^b^	1.98 ± 0.09 ^c^	1.92 ± 0.20 ^c^	2.28 ± 0.09 ^b c^	2.45 ± 0.12 ^b^
	7	7.01 ± 0.14 ^a^	2.48 ± 0.46 ^b^	1.84 ± 0.18 ^b^	2.05 ± 0.02 ^b^	2.02 ± 0.17 ^b^	2.19 ± 0.22 ^b^
	8	7.28 ± 0.32 ^a^	2.70 ± 0.10 ^b^	1.78 ± 0.16 ^c^	1.90 ± 0.13 ^c^	1.98 ± 0.03 ^c^	2.17 ± 0.26 ^c^
	9	6.65 ± 0.43 ^a^	3.16 ± 0.05 ^b^	1.83 ± 0.07 ^c^	2.13 ± 0.13 ^c^	1.98 ± 0.19 ^c^	2.14 ± 0.25 ^c^
	10	7.60 ± 0.35 ^a^	2.98 ± 0.09 ^b^	1.82 ± 0.07 ^c^	1.87 ± 0.07 ^c^	2.04 ± 0.04 ^c d^	2.48 ± 0.10 ^d^
	12	7.76 ± 0.19 ^a, 2^	3.38 ± 0.31 ^b, 2^	1.76 ± 0.13 ^c, 1^	1.57 ± 0.18 ^c, 1^	1.94 ± 0.47 ^c, 1^	2.18 ± 0.22 ^c, 1^

Description of cooked ham samples: P1: Negative control; P2: 100 ppm nitrite + 250 ppm ascorbic acid + 0.7% PRS-DV-5; P3: 1% NATPRE T-10 DV HS + 0.5% PRS-DV-5; P4: 1% NATPRE T-10 DV LS + 0.5% PRS-DV-5 LS + 1.3% NaCl + 0.35–0.40% KCl; P5: 1% NATPRE T-10 EML+ 0.5% PRS-DV-5; P6: 1% NATPRE T-10 EML + 0.75% PRS-DV-5. Statistical analysis: letters a–d: Different letters within the same row indicate significant differences between samples in the same analysis time (*p* < 0.05); Numbers 1–2: Different numbers within the same column in the 12th week between temperatures (*p* < 0.05).

**Table 4:** Growth potential parameters obtained after different storage times and temperatures for the different cooked ham samples (P1–P6).

Temperature	Week	P1	P2	P3	P4	P5	P6
4 °C	4	3.65	−0.22	−0.51	−0.46	−0.50	−0.64
	5	3.00	−0.36	−0.54	−0.37	−0.21	−0.31
	7	3.56	0.01	−0.47	−0.33	−0.28	−0.46
	8	3.92	−0.18	−0.37	−0.28	−0.10	−0.21
	10	4.07	−0.14	−0.21	−0.21	−0.13	−0.23
	12	3.49	0.13	−0.35	−0.16	−0.20	−0.17
	13	3.35	−0.12	−0.66	−0.31	0.08	−0.32
	14	3.96	−0.17	−0.58	−0.33	−0.18	−0.31
	15	5.56	−0.12	−0.49	−0.39	−0.09	−0.20
	16	5.32	−0.30	−0.34	−0.44	−0.26	−0.47
	17	5.31	−0.39	−0.52	−0.36	−0.10	−0.13
	δ maximum	5.56	0.13	−0.21	−0.16	0.08	−0.13
7 °C	3	4.43	0.28	−0.35	−0.24	0.05	−0.11
	4	4.60	0.21	−0.43	−0.23	−0.24	−0.41
	5	4.98	0.33	−0.30	−0.25	0.04	−0.12
	7	4.97	0.59	−0.44	−0.12	−0.22	−0.38
	8	5.23	0.81	−0.50	−0.27	−0.26	−0.40
	9	4.61	1.27	−0.45	−0.04	−0.26	−0.43
	10	5.56	1.09	−0.46	−0.30	−0.20	−0.09
	12	5.72	1.48	−0.52	−0.60	−0.30	−0.39
	δ maximum	5.72	1.48	−0.21	−0.04	0.08	−0.09

Description of cooked ham samples: P1: Negative control; P2: 100 ppm nitrite + 250 ppm ascorbic acid + 0.7% PRS-DV-5; P3: 1% NATPRE T-10 DV HS + 0.5% PRS-DV-5; P4: 1% NATPRE T-10 DV LS + 0.5% PRS-DV-5 LS + 1.3% NaCl + 0.35–0.40% KCl; P5: 1% NATPRE T-10 EML+ 0.5% PRS-DV-5; P6: 1% NATPRE T-10 EML + 0.75% PRS-DV-5.

## Data Availability

The data used to support the findings of this study can be made available by the corresponding author upon request.
